# Factors associated with exclusive breastfeeding practice in a cohort of women from Cali, Colombia.

**DOI:** 10.25100/cm.v50i1.2961

**Published:** 2019-03-30

**Authors:** Julio Cesar Mateus Solarte, Gustavo Alonso Cabrera Arana

**Affiliations:** 1 Universidad del Valle, Facultad de Salud, Escuela de Salud Pública, Cali, Colombia; 2 Universidad de Antioquia, Facultad Nacional de Salud Pública, Medellin, Colombia

**Keywords:** Breast feeding, infant nutrition, Colombia, duration of exclusive breast feeding, cohorts, maternal health, milk human, child nutrition disorder, fathers, postpartum period, Lactancia materna, nutrición del lactante, Colombia, duración de lactancia exclusiva, cohortes, salud materna, leche humana, desordenes en nutrición infantil, padres, periodo postparto

## Abstract

**Introduction:**

Breastfeeding promotion is one of the most effective strategies to prevent child malnutrition; it reduces costs to families, health services and society. In Colombia, exclusive breastfeeding is practiced only by 10% of women.

**Objective:**

To identify factors associated with the duration of exclusive breastfeeding.

**Methods:**

A cohort of 438 primiparous women was followed during 6 months by means of 8 home interviews, in order to determine the duration of exclusive breastfeeding. Individual, family and health service factors were studied; and survival analysis was carried out.

**Results:**

At 8 days, only a few more than half of the participants maintained exclusive breastfeeding; at month 6 of follow-up, this proportion was reduced to 1.4%. The duration of exclusive breastfeeding was determined by: initiation of breastfeeding in the first 4 hours after delivery (HR= 4.07, 95% CI: 0.96-16.67), self-perceived sureness for breastfeeding (HR= 1.28, 95% CI: 1.04 -1.58), positive opinion of the baby's father regarding breastfeeding (HR= 1.26, 95% CI: 1.01-1.57), and newborn weight (HR= 1.23, 95% CI: 1.00-1.53).

**Conclusion:**

There are factors before delivery and in the immediate puerperium that determine, partially, the duration of exclusive breastfeeding.

## Resumen

**Introducción:**

La promoción de la lactancia es una de las estrategias más efectivas para evitar la desnutrición infantil; reduce costos a las familias, los servicios de salud y la sociedad. En Colombia solo 10% de las mujeres practican la lactancia materna exclusiva.

**Objetivo:**

Identificar factores asociados a la duración de la lactancia exclusiva.

**Métodos:**

Se siguió durante 6 meses a una cohorte de 438 mujeres primíparas mediante 8 entrevistas domiciliarias para determinar la duración de lactancia exclusiva. Se estudiaron factores individuales, familiares y de servicios de salud y se realizó análisis de sobrevida.

**Resultados:**

A los 8 días sólo un poco más de la mitad de participantes mantenía lactancia exclusiva; al mes 6 de seguimiento esta proporción se redujo a 1.4%. La duración de lactancia exclusiva estuvo determinada por: inicio de lactancia en las primeras 4 horas pos-parto (HR= 4.07; IC 95%: 0.96-16.67), seguridad auto-percibida para amamantar (HR= 1.28; IC 95%: 1.04-1.58), opinión positiva del padre del bebé frente a lactancia (HR= 1.26; IC 95%: 1.01-1.57) y peso de recién nacido (HR= 1.23; IC 95%: 1.00-1.53).

**Conclusión:**

Existen factores antes del parto y en el puerperio inmediato que determinan, parcialmente, la duración de la lactancia exclusiva.

## Introduction

Malnutrition can begin in utero and spread throughout the life cycle of people, and even the generational cycle of families, communities or nations ^1^. Under these conditions, poor nutrition can occur in the prenatal or postnatal stage, which increases the risk of illness and death in infants, and negatively impacts the development and well-being of future adolescents and adults ^2^ ^-^ ^4^. There is evidence showing the protection provided by breastfeeding against the risk of death and infectious diseases in early childhood ^2^ ^,^ ^5^. Benefits in child neurodevelopment, maternal health and the prevention of chronic adult diseases have been demonstrated ^4^ ^,^ ^6^ ^-^ ^11^. Consequently, the promotion and protection of breastfeeding is considered one of the most effective strategies to reduce malnutrition in young children ^1^ **.**

In addition, it has been determined that breast milk, as the only food, can provide all the necessary nutrients for a child aged 6 months, or younger, in order to have a proper development; and that from this age, if complemented with other foods, it may keep providing important nutrients until two years of life ^12^.

Despite this, the practice of exclusive breastfeeding (LME, for its initials in Spanish) during the first six months continues to be low in many regions of the world. In Colombia, according to the National Survey of Demography and Health, between 2005 and 2010 the median of duration of LME decreased from 2.2 to 1.8 months; and the proportion of women who practiced it decreased from 46.8% to 43.7% ^13^ ^,^ ^14^. Similarly, in Cali, a prospective study determined that the median of duration of LME was 11 days, and that only 1.4% of women practiced it as recommended ^15^.

In many communities, it has been identified that the practice and duration of LME is conditioned by a series of individual, family, health and contextual services factors, whose relative importance is variable across communities ^16^ ^-^ ^19^. Consequently, it has been observed that standardized interventions to promote the practice and increase the duration of LME do not reach the same level of effectiveness when they are implemented in different communities ^20^ ^-^ ^22^. For this reason, determining the relative importance of the factors that determine LME is essential, in order to promote its practice and increase its duration in a particular community. This study will take as reference the Theory of Planned Behavior (TPB), since it has been widely used to assist in the identification of the possible factors involved in the execution of a desirable behavior for health, among them breastfeeding ^19^, and to provide elements to identify the possible causal pathways of the identified factors ^23^.

The objective of this study was to identify individual, family and health services factors that limit or promote the duration of LME in Cali, Colombia. This was aimed at generating knowledge that would allow strengthening the promotion and protection of LME in this city.

## Materials and Methods

### Type of study

Study of a fixed cohort of women, in immediate puerperium, whose recruitment was done in health institutions in the first four postpartum hours. The follow-up was done in eight home visits at 8, 15, 30, 60, 90, 120, 150 and 180 postpartum days. At each visit, it was established whether the woman was exclusively breastfeeding or not. The follow-up ended when LME was abandoned, when the newborn died, when the woman could not be located for the subsequent visits, or when there were reached 180 days of LME.

### Population

We studied primiparous women living in Cali, Colombia, with delivery attended in six state or private institutions, of levels I or II of complexity, who ended their pregnancy between 37 and 40 weeks of gestation, with a single newborn whose weight ranged between 2,500 and 4,500 g; both the mothers and their newborns did not require hospitalization for more than 24 hours, and who voluntarily accepted to enter the study at four postpartum hours. Primiparous women were selected because they represent almost 60% of those who start breastfeeding in the city.

### Sample

According to previous studies in Colombia and other countries ^18^ ^,^ ^24^ ^-^ ^27^, the proportion of women who practice LME progressively decreases in the first postnatal months. Taking into account this behavior, and that when arriving at the beginning of the first six months (151 days) only 10% of Colombian women still practice LME, it was established that, with a minimum hazard ratio of 2, power of 80 %, and 95% confidence, 438 women were needed for this study.

### Ethical considerations

This research was approved by the ethics committee of the FES Social Foundation, in accordance with guidelines outlined in a call of the National Program of Health Science and Technology of COLCIENCIAS, the Declaration of Helsinki and Resolution 008430 of 1993 of the Ministry of Health from Colombia.

### Definition of variables

The studied variables responded to the identification of factors that can potentially limit or promote the duration of LME, based on the theory of planned behavior (TPB) and characteristics of the provision of health services offered during pregnancy and the immediate postpartum period. The outcome variable was the time in days elapsed from entry into the study until the abandonment of LME. LME was considered if the infants received only breast milk, or when they received LME along with a limited amount of water that did not alter the usual frequency of breastfeeding (16 cc or less, twice a day), regardless of whether they took it directly from the breast or from an artificial container. We considered abandonment of LME when a baby received more than 32 cc of water per day, or other solid or semisolid foods different from breast milk.

The independent variables measured demographic and social characteristics, beliefs, attitudes and practices related with breastfeeding, pregnancy planning, prenatal care attendance, maternal intention to breastfeed (duration and sureness), joint accommodation, early initiation of breastfeeding, frequency of reported breastfeeding, perception about close family references, type of affiliation to the Colombian health system, quality and quantity of information about breastfeeding received during pregnancy and the immediate postpartum period from health personnel, use of utensils to feed the newborn, perception of the father regarding breastfeeding, and variables of the newborn.

A particular aspect in the Colombian context is the changes introduced in the provision of services due to a reform of the health system ^28^. This reform created two regimes of affiliation: a contributory regime, whose contributors are employed people; and a subsidized regime, for people who do not have a job; it also defined mechanics of financing and care for the population that was not able to join any of the regimes; and established that the services offered to each type of affiliation must be of the same characteristics ^29^. In addition, there were created insurance institutions called Benefit Plans Administering Entities, and Health Service Provider Institutions. The former are obliged to structure networks of services for the attention of their members; for this, they establish contracts with the Health Service Provider Institutions. For this reason, the type of affiliation was incorporated as a variable of interest for the study.

### Collection of information

The recruitment was carried out in six health institutions with basic and intermediate care, which attended 80% of the annual births in Cali. Informed consent was requested, and the collection started with a face-to-face interview when the women were still in the institution, during the first four hours postpartum. The gestational age and weight of the newborn were obtained from the clinical history. The instrument design was based on previous instruments, which were adjusted taking into account the results of a pre-test, whose objective was to improve the understanding and intention of the questions.

### Statistical analysis

The univariate analysis determined the frequency and distribution of variables. The bivariate explored the correlation between variables and their relation to the duration of LME. Then, a descriptive and bivariate survival analysis was performed in order to obtain the survival function according to strata of each independent variable. Through the Log Rank Test, it was established if this function presented significant differences; with this analysis, it was determined which variables were related to the duration of LME at levels of significance of *p* ≤0.25; and with these, we proceeded to construct the multiple model. Unadjusted and adjusted associations were estimated among the independent variables and the dependent one (time at abandonment of LME) using the Cox regression. We assessed confusion and possible modifications of the effect on the relation that each variable incorporated in the multiple analysis had with the duration of LME. 

## Results

During 75 consecutive days, 453 puerperae were invited to participate; 15 rejected to do it. A unique cohort of 438 women was formed. After the initial interview, 24 women (5.5%) could not be located for the first home visit on the 8th day at the address or the reported telephone number, so they were declared as losses. Two more women (0.5%) suffered the death of their babies before the first visit. Between the first and the fifth visit, there were 12 additional losses (2.7%), the majority due to unreported changes of address of the participants. The total loss to follow-up was 38 women (8.7%). The majority of the participating women were aged between 15 and 24 years, living in free union, with high school studies, and dedicated to their homes (housewives). The distribution of the type of affiliation was similar among the participants (Table 1).

**Table 1:** Socio-demographic characteristics of women from the Cali cohort.

Characteristic	n	%
Age in years		
< 15	16	3.7
15-24	362	82.6
> 24	60	13.7
Civil status		
Single	144	32.9
Married	29	6.6
Free Union	263	60.0
Other	2	0.5
Education (school) level		
None	3	0.7
Primary	70	16.0
Secondary/High School	317	72.4
Technical/University*	48	11.0
Occupation		
Home (housewife)	323	73.7
Working	52	11.9
Studying	18	4.1
Two or more	42	9.6
Type of affiliation to the Colombian health system		
Contributory regime	144	33.0
Subsidized regime	160	36.6
Non-affiliated	133	30.4

*The technical/university education (school) level was investigated as a single category

A few more than half of newborns were male (50.5%), birth weight had a normal distribution with an average of 3,297 g (95% CI: 3,173-3,241) and the vaginal route was the most used in childbirth (n= 323, 73.7%). Regarding conception and pregnancy, 267 (61.0%) of the participants said they did not expect to become pregnant; 344 (78.5%) of them learnt of their pregnancy before the third month of pregnancy, and 427 (97.5%) reported having attended prenatal care. 254 (58.0%) of the participants did not receive any orientation from health personnel about LME during pregnancy and, between birth and study entry, 283 (64.6%) had not received any orientation either. The proportion of women in the contributory regime who received orientation from health personnel was greater than the proportion of affiliates to the subsidized one, and also greater than the proportion of unaffiliated women (Table 2).

**Table 2:** Proportion of women with orientation in breastfeeding by health personnel, according to affiliation to the General Social Security System.

Affiliation	n	Orientation during puerperium (%)*	Orientation during pregnancy (%)**
Contributory regime	144	36.8	42.4
Subsidized regime	160	34.8	32.1
Non-affiliated women	133	28.4	25.5

*X^2^_gl=2_=1.60; *p*= 0.449

** X^2^_gl=2_=12.89; *p*= 0.002

Most of the women reported not knowing how to keep the extracted milk at home. There was also a significant lack of knowledge and basic skills to maintain successful breastfeeding. With the exception of the technique to place the baby to the breast, women reported less deficit in knowledge about breastfeeding frequency and greater deficits in breast milk extraction, milk conservation, and duration of breastfeeding at the time of immediate puerperium, when it is imminent the onset of breastfeeding; these differences had statistical significance (Table 3).

**Table 3:** Frequency of knowledge and basic skills to maintain successful breastfeeding in pregnancy and puerperium in the cohort.

Deficit in knowledge and basic skills for breastfeeding	Pregnancy		Immediate puerperium		p*
	n	%	n	%	
Technique to place the baby to the breast	57	13.0	65	14.8	0.50
Breast milk extraction	119	27.2	198	45.2	0.00
Breast milk conservation at home	195	44.6	364	83.2	0.00
Duration of breastfeeding	124	28.3	265	60.6	0.00
Frequency of breastfeeding	145	33.2	138	31.6	0.02

* McNemar test

**Figure 1:**
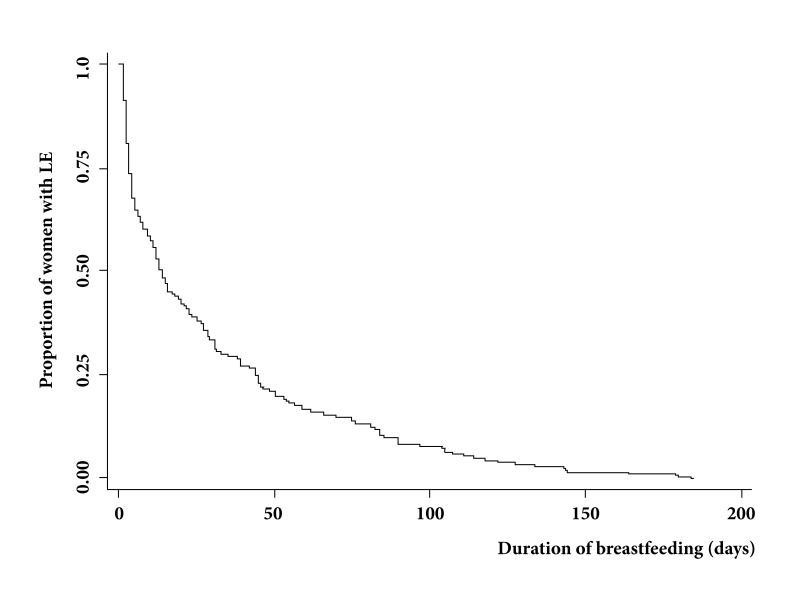
Survival of exclusive breastfeeding in breastfeeding primiparous women from Cali, Colombia.

The majority (n= 284, 64.8%) reported not having spoken with the father of the baby about LME; among 154 (35.2%) who did it, the father's opinion was positive in 150 (98%). In the first interview, the majority (n= 415, 94.7%) did not know the norm or law that protected breastfeeding. Joint accommodation was observed in 92.9%. Of 438 women, only 133 (30.4%) had intention of LME for 6 months, 82 (18.8%) for less than 6 months, 64 (14.6%) for more than 6 months, and 158 (36.2%) did not know for how long. Of 437 who expressed intention to breastfeed, 72.9% said they were very sure of doing so, 26.7% said they were moderately sure, and 0.4% said they were not sure. When entering the follow-up, 432 (98.6%) were already breastfeeding their baby, 386 (89.4%) at free requirement, and 175 (40.5%) had begun breastfeeding in the first half hour postpartum. Among 185 women with difficulties to breastfeed, 52 (28.1%) said that they had low milk supply, 43 (23.2%) pain in their breasts, and 52 (28.1%) did not know how to place the baby. Figure 1 shows the progressive and marked decrease in the proportion of women who maintained LME as the follow-up progressed. At 8 days, a few more than half of them kept LME; and at the end of the follow-up, this proportion was reduced to only 1.4%.

It was observed that the incidence rate of LME abandonment in the cohort was 33.77 (95% CI: 30.50-37.39) dropouts per 1,000 women-days of LME. At 3 days (95% CI: 3.0-4.0), 25% had already left LME; at 11 days (95% CI: 7.1-13.0), 50% had already done so; and at 44 days (95% CI: 35.0-48.0), 75% had left LME. It was determined that only four variables among the studied ones were associated with duration of LME, with level of significance *p* ≤0.25; and these were the ones selected for multiple analysis. It was found that women who were already breastfeeding in the first 4 hours postpartum had a significantly longer duration of LME than those who did not. Women who expressed being very sure to breastfeed their babies kept LME for longer than those who expressed being moderately sure or (those) unsure to do so. When the baby's father had a positive opinion about breastfeeding, the duration of LME was significantly longer than when he did not have it, or he had not had an opinion on the subject. It was found that in the range of birth weight that was studied, the relationship with the duration of LME was not linear. Since in the weight ranges between 2,500 - 3,021 g and 3,388-4,500 g, the duration of LME was greater than when the newborn weighed between 3,022 and 3,387, the weight of the newborn was categorized taking into account this relationship (Table 4).

**Table 4:** Variables that influenced the duration of exclusive breastfeeding.

Variable	n	Median of breastfeeding in days	IC 95%	Log Rank Test	*p*
Sureness (for providing breastfeeding)					
Very sure	199	17	13-27	7.29	0.007
Moderately or insecure	239	12	7-15		
Positive opinion of baby's father on breastfeeding					
Yes	150	17	12-26	4.81	0.028
Negative/He didn't have an opinion	288	12	9-16		
Weight at birth					
≤3,021 or >3,387 g	274	15	12-21	4.67	0.031
3,022 a 3,387 g	164	11	7-16		
Breastfeeding while in puerperium					
Yes	432	14	12-17	6.95	0.008
No	6	2	-		

The association of the variable that indicates whether during the immediate puerperium, the mother was breastfeeding or not, and the duration of LME, becomes statistically insignificant during the multiple analysis. However, despite a greater than 10% change between the unadjusted and adjusted estimates for this variable, there was not shown that the change had been influenced (confusion or effect modification) by the other variables associated with duration of LME. The other unadjusted and adjusted hazard ratios do not differ significantly. Thus, it is plausible that the relationship of each variable associated with LME duration is independent, and is not influenced by other associated variables. Consequently, it would be expected that if it is implemented an intervention to increase breastfeeding sureness in women of similar characteristics to those studied here, the risk of abandoning exclusive LME early could be significantly reduced by 28%. Similarly, if a greater proportion of fathers, similar to the partners of the studied women, have a positive opinion of breastfeeding, it would be expected that the risk of leaving LME early would be significantly reduced by up to 26%. Finally, if it is possible to effectively promote LME in newborns of women similar to those studied here, whose weights range between 3,022 and 3,387 grams, it would be expected that the risk of early LME abandonment would be significantly reduced by 23%. (Table 5)

**Table 5:** Unadjusted and adjusted hazard ratios for abandoning exclusive breastfeeding

Variable	Unadjusted hazard ratios	CI 95%	Adjusted hazard ratios	CI 95%
Sureness (for providing breastfeeding)				
Very sure	1.00	-	1.00	-
Moderately or insecure	1.32	1.07-1.62	1.28	1.04-1.58
Positive opinion of baby's father on breastfeeding				
Yes	1.00	-	1.00	-
Negative/He didn't have an opinion	1.27	1.02-1.58	1.26	1.01-1.57
Weight at birth				
≤3,021 or >3,387 g	1.00	-	1.00	-
3,022 a 3,387 g	1.26	1.02-1.56	1.23	1.00-1.53
Breastfeeding in immediate puerperium				
Yes	1.00	-	1.00	-
No	5.01	1.23-20.43	4.07	0.96-16.67

## Discussion

This was a study in a single, fixed cohort of breastfeeding women living in Cali, which reached its goal of identifying possible causal factors of LME duration. This design allowed the measurement of the duration of LME in a prospective way, and not a retrospective one, as in most of the national studies. Prospective measurement confers advantages since studies that retrospectively measure the duration of LME introduce information biases that have a high probability of affecting the validity of results ^30^ ^-^ ^32^. The differences in the estimation of the duration of LME and the proportion of women who practice LME of this study with the National Survey of Demography and Health in Colombia ^14^ are probably due not only to the difference that may exist in behavioral factors and/or socio-cultural and health service provision between Cali and the rest of the country, but also to the validity of the methods used in the measurements. The National Survey of Demography and Health estimates the duration of LME and the proportion of women who practice it using retrospective techniques that seek to evoke recall in women who breast-fed several years ago, which affects the validity of the estimate ^32^.

In this study, it was found that most women expressed a firm intention to breastfeed in the immediate puerperium, but the proportion who finally breast-fed their babies during the first six months was low; very few provided LME during the recommended time. This finding questions one of the fundamental assumptions of the TPB, which states that the intention to execute a behavior is a good predictor of its execution. However, it could be possible the need to improve the quest for intent to breastfeed.

This study also showed that factors present before delivery or the immediate puerperium are independently involved in the duration of LME. The effect of the initiation of breastfeeding in the immediate puerperium on the duration of LME has already been found in other investigations. The mechanism by which this factor is involved in the duration of LME is apparently because early suction favors the second stage of lactation, or lactogenesis (creation of milk); and it is when the continuous production of milk is established, as well as the capacity to respond to demands of the newborn. This variable shows a significant association in the bivariate analysis (*p*= 0.008), which subsequently becomes non-significant in the multiple analysis, possibly due to the low frequency of women who did not initiate early breastfeeding. However, despite the wide confidence interval, it is marginally included the null value of the adjusted hazard ratio. This situation does not rule out the effect of this variable on LME in Cali; and for future studies, it should be kept in mind since it can produce adjustments in the relationships of other variables with LME (*p* <0.25 in bivariate), if the number of women who do not start early LM increases, as it can be expected if the trends of duration and practice of LME reported by national surveys are valid.

This study found that the self-perception of sureness regarding the initiation and maintenance of LME in the immediate puerperium is associated with its duration ^16^ ^,^ ^33^. This information obtained from primiparous women residing in Cali, with no practical experience in breastfeeding, suggests that there are factors of the immediate puerperium implicated in the causality of the duration of LME. According to TPB, moderate or little self-perceived sureness triggers a succession of events that ultimately do not favor the practice of LME. In the first place, low self-perception induces in women the feeling that they will not control breastfeeding and, therefore, they will not be able to overcome the barriers to breastfeeding, or to use conditions that facilitate it. Consequently, the intention to breastfeed is weakened, breast milk substitutes are more easily used, and LME is interrupted. 

However, it has been found that as breastfeeding progresses, the sureness to breastfeed in primiparous women increases until reaching the same levels of multiparous women. This is a possible mechanism by which the social support network (especially the baby's father and grandmothers) prolongs the duration of breastfeeding. The effect of the opinion of the baby's father on the duration of LME found in this study coincides with the results of other studies ^20^ ^,^ ^34^, and places them as one of the potential participants that could support the extension and maintenance of LME in populations similar to the one studied here. From the TPB, the positive opinion of the father raises the perception that many people approve LME in women, since it establishes in them the feeling that this immediate referent approves LME, which increases the motivation to please him. Thus, it is reinforced the intention to provide LME and effectively practice it for a longer time. 

Consequently, when the father of the newborn is trained to provide better support to the woman who breastfeeds, LME is prolonged for longer, and there increases the proportion of women who practice it ^20^ ^,^ ^34^.

The relationship between birth weight and duration of LME found here, partially agrees with the results of prospective studies ^16^ ^,^ ^35^ ^,^ ^36^. It agrees in that it continues to be observed that children of lower birth weight are the ones who receive LME for a longer time. But the fact that newborns weighing more than 3,487 g receive LME for a longer time is a particular finding. One possible explanation is that the health professionals who attended those women gave greater theoretical-practical support in breastfeeding to women whose newborns weighed 3,021 or less, or when they weighed more than 3,387 g, in order to have a faster weight gain in the former; and in order to avoid a potential hypoglycemia in the latter. This association must be addressed in later studies.

The information and support provided during pregnancy and the puerperium were not involved in the duration of LME. It is possible that in a population like the one here studied, in which the duration of LME is so short and practiced for 6 months by so few women, the high level of ignorance in basic aspects does not present a sufficient gradient to establish differences. Another possibility is that during the course of breastfeeding, the knowledge deficit is corrected or new factors arise, or the factors present in the pregnancy or in the puerperium are modified, and these are the ones that explain the abandonment of LME. This last possibility cannot be supported with this study because the methodology focused on factors present in pregnancy and in the immediate puerperium, and the evolution that these factors could have had during the follow-up was not measured. This weakness must be addressed in later prospective studies.

Factors such as maternal education, socio-economic stratum, occupation, marital status and type of birth, variables that have traditionally been involved at the time of LME, were not involved in the duration of LME in Cali. This reflects homogeneity in the distribution of these socio-economic variables between women who abandoned LME early, and those who did not. With regard to the way of delivery, it is possible that it was not involved in the duration of LME in this population, because in the great majority of the births, it was vaginal (73.7%), and almost all women and their babies had joint accommodation and started breastfeeding early, counteracting the effect of caesarean section.

The mother-child binomials affiliated to the Health System regimes did not show differences in duration of LME with respect to the non-affiliated binomials. However, the proportion of women who received counseling during pregnancy was significantly lower in affiliates to the subsidized regime and in those not affiliated, in comparison with affiliates to the contributory regime. This suggests inequalities in the provision of services according to the type of affiliation. This finding suggests little effectiveness of the Health System to promote breastfeeding because, despite offering more education and support to women affiliated to the contributory regime, it does not achieve a longer duration than that observed in women of the subsidized regimes, or in non-affiliated women.

## Conclusions

There are individual, family and health services factors that are present before and immediately after delivery, which limit the duration of LME in Cali.

The use of the TPB was convenient to identify possible causal factors of the short duration of LME in Cali, and it provided a useful guide on the possible pathways through which its effects are triggered.

**Recommendations**. It is a priority to raise awareness and to improve practices on basic aspects of breastfeeding, not only in pregnant women or during puerperium, but on the most significant and influential people for these women, such as the fathers of the newborns. In addition, there should be reinforced the skills of health staff.

Sureness for breastfeeding, the opinion that the father of the newborn has of breastfeeding, and birth weight can be used in three ways to address the short duration of LME. In the first place, modifying these factors should be the objective of the interventions that are implemented from the immediate puerperium. Secondly, the factors identified can help to detect in the immediate puerperium the women at greater risk of early abandonment of LME, and initiate a timely intervention in them. Finally, the measurement of the duration of LME in this study can serve as a reference to compare the effectiveness of different intervention alternatives aimed at prolonging the duration of LME. 
