# Supplementary material for: Factors associated with exclusive breastfeeding practice in a cohort of women from Cali, Colombia
Source: Colomb Med (Cali). 2019 Mar 30;50(1):22–9. doi: 10.25100/cm.v50i1.2961 (PMC6536044; doi:10.25100/cm.v50i1.2961)
Supplement: Related texts

# Original article: Factores asociados con la práctica de lactancia exclusiva en una cohorte de mujeres de Cali, Colombia

## Introducción

La desnutrición puede iniciarse in útero y extenderse a lo largo del ciclo vital de las personas, e incluso del ciclo generacional de familias, comunidades o naciones ^1^. Bajo estas condiciones se puede presentar una nutrición deficiente en la etapa prenatal o postnatal que eleva el riesgo de enfermar y morir en los infantes e impacta negativamente el desarrollo y bienestar de futuros adolescentes y adultos ^2^^-^^4^. Existe evidencia que demuestra la protección que brinda la lactancia materna frente al riesgo de muerte y de enfermedades infecciosas en la infancia temprana ^2^^,^^5^. Se han demostrado beneficios en el neurodesarrollo infantil, la salud materna y la prevención de enfermedades crónicas de la adultez ^4^^,^^6^^-^^11^. En consecuencia, la promoción y protección de la lactancia es considerada una de las estrategias más efectivas para disminuir la desnutrición del infante menor ^1^.

Adicionalmente se ha determinado que la leche materna, como único alimento, puede brindar todos los nutrientes necesarios para que un niño de 6 o menos meses se desarrolle adecuadamente y, que a partir de esta edad, si se complementa con otros alimentos, puede continuar brindando nutrientes importantes hasta los dos años de vida ^12^. 

A pesar de esto, la práctica de la lactancia materna exclusiva (LME) durante los primeros seis meses continúa siendo baja en muchas regiones del mundo. En Colombia, según la Encuesta Nacional de Demografía y Salud, entre 2005 y 2010 la mediana de duración de LME disminuyó de 2.2 a 1.8 meses y la proporción de mujeres que la practicaron decreció de 46.8% a 43.7% ^13^^,^^14^. De la misma manera en Cali un estudio prospectivo determinó que la mediana de duración de LME fue de 11 días y que solo 1.4% de las mujeres la practicaron según lo recomendado ^15^. 

En muchas comunidades se ha identificado que la práctica y la duración de LME esta condicionada por una serie de factores individuales, familiares, de los servicios de salud y contextuales, cuya importancia relativa es variable a través de las comunidades ^16^^-^^19^. En consecuencia, se ha observado que intervenciones estandarizadas para promover la practica y aumentar la duración de LME no alcanzan el mismo nivel de efectividad cuando son implementadas en comunidades diferentes ^20^^-^^22^. Por tal motivo, determinar la importancia relativa de los factores que determinan LME es indispensable para promover su práctica y aumentar su duración en una comunidad particular. Este estudio tomará como referencia la Teoría del Comportamiento Planeado (TCP), dado que ha sido ampliamente utilizada para asistir en la identificación de los posibles factores involucrados en la ejecución de un comportamiento deseable para la salud, entre ellos lactancia ^19^, y brindar elementos para identificar las posibles vías causales de los factores identificados ^23^. 

El objetivo de este estudio fue identificar factores individuales, familiares y de los servicios de salud que limitan o promueven la duración de la LME en Cali. Con esto se buscó generar conocimiento que permitiera fortalecer la promoción y protección de la LME en la ciudad.

## Materiales y Métodos

### Tipo de estudio

Estudio de cohorte fija de mujeres en puerperio inmediato, cuyo reclutamiento se hizo en instituciones de salud en las primeras cuatro horas posparto. El seguimiento se hizo en ocho visitas domiciliares a los 8, 15, 30, 60, 90, 120, 150 y 180 días posparto. En cada visita se estableció si la mujer estaba lactando en forma exclusiva o no. El seguimiento finalizó cuando ocurría el abandono de la LME, cuando fallecía el recién nacido, cuando la mujer no pudo ser localizada para las visitas subsiguientes o cuando se llegaba a 180 días de LME. 

### Población

Se estudiaron mujeres primíparas residentes en Cali, Colombia, con parto atendido en seis instituciones públicas o privadas de nivel I y II de complejidad, que terminaron embarazo entre 37 y 40 semanas de gestación, con un solo recién nacido cuyo peso osciló entre 2,500 y 4,500 g, que tanto ellas como su recién nacido no requirieron hospitalización por más de 24 horas y aceptaron voluntariamente ingresar al estudio en las cuatro horas posparto. La selección de mujeres primiparas se hizo porque representan casi 60% de las que inician lactancia en la ciudad. 

### Muestra

Según estudios previos en Colombia y otros países ^18^^,^^24^^-^^27^, la proporción de mujeres que practica LME disminuye progresivamente en los primeros meses posnatales. Teniendo en cuenta este comportamiento, y que al llegar al inicio de los seis primeros meses (151 días) solo 10% de mujeres colombianas aun practican la LME, se estableció que, con una razón mínima de peligro a detectar de 2, poder de 80%, confianza de 95%, eran necesarias 438 mujeres en el estudio.

### Consideraciones éticas

Esta investigación fue aprobada por el comité de ética de la Fundación FES Social, de acuerdo con lineamientos señalados en una convocatoria del Programa Nacional de Ciencia y Tecnología de la Salud de COLCIENCIAS, la Declaración de Helsinki y la Resolución 008430 de 1993 del Ministerio de Salud de Colombia.

### Definición de variables

Las variables estudiadas respondieron a la identificación de factores que potencialmente pueden limitar o promover la duración de la LME tomando como base la teoría del comportamiento planeado (TCP) y características de la prestación de los servicios de salud ofrecidos en el embarazo y el puerperio inmediato. La variable resultado fue el tiempo en días transcurrido desde el ingreso al estudio hasta el abandono de la LME. Se consideró LME si el infante recibía únicamente leche materna o cuando recibía LME junto con una cantidad limitada de agua que no alteraba la frecuencia habitual del amamantamiento (16 cc o menos, dos veces al día), sin importar si la tomaba directamente de la mama o de un recipiente artificial. Se consideró abandono de la LME cuando el infante recibió alimentos diferentes a la leche materna o más de 32 cc de agua al día u otros alimentos sólidos o semisólidos.

Las variables independientes midieron características demográficas, sociales, creencias, actitudes y prácticas frente a lactancia materna, planificación del embarazo, asistencia a control prenatal, la intención materna para amamantar (duración y seguridad), el alojamiento conjunto, inicio temprano de lactancia, frecuencia de amamantamiento reportada, percepción sobre referentes familiares cercanos, tipo de afiliación al sistema de salud, calidad y cantidad de información sobre lactancia recibida durante el embarazo y el puerperio inmediato del personal de salud, uso de utensilios para alimentar al recién nacido, percepción del padre frente a la lactancia, y variables del recién nacido.

Un aspecto particular en el contexto colombiano son las modificaciones introducidas en la prestación de servicios por una reforma al sistema de salud ^28^. Esta reforma creó dos régimenes de afiliación, un régimen contributivo cuyos aportantes son las personas con empleo, un régimen subsidiado para las personas que no tienen empleo y definió mecánismos de financiación y atención para la población que no lograba afiliarse a alguno de los regimenes. Además estableció que los servicios ofrecidos a cada tipo de afiliación deben ser de iguales características ^29^. También se crearon instituciones aseguradoras denominadas Entidades Administadoras de Planes de Beneficio e Instituciones Prestadoras de Servicios de Salud. Las primeras están obligadas a estructurar redes de servicios para la atención de sus afiliados y para ello establecen contratos con las Instituciones Prestadoras de Servicios. Por esta razón, se incorporó el tipo de afiliación como una variable de interés para el estudio. 

### Recolección de la información 

El reclutamiento se realizó en seis instituciones de salud con atención básica e intermedia que atendían 80% de los partos anuales en Cali. Se solicitó consentimiento informado y la recolección se inició con una entrevista cara-cara cuando la mujer aún estaba en la institución, en las primeras cuatro horas posparto. La edad gestacional y el peso del recién se obtuvieron de la historia clínica. El diseño de instrumentos partió de instrumentos previos, que se ajustaron teniendo en cuenta los resultados de una pre-prueba cuyo objetivo fue mejorar la comprensión e intención de las preguntas.

### Análisis estadísticos

El análisis univariado determinó la frecuencia y distribución de variables. El bivariado exploró la correlación entre variables y su relación con la duración de la LME. Luego se realizó un análisis de sobrevida descriptivo y bivariado, para obtener la función de sobrevida según estratos de cada variable independiente. Mediante el Log Rank Test se estableció si dicha función presentaba diferencias significantes; con este análisis se determinó cuáles variables se relacionaron con la duración de la LME a niveles de significancia de *p* (0.25 y con estas se procedió a construir el modelo múltiple. Se estimaron asociaciones crudas y ajustadas entre variables independientes y la dependiente (tiempo al abandono de LME) utilizando la regresión de Cox. Se evaluó confusión y posibles modificaciones del efecto en la relación que cada variable incorporada al análisis múltiple tuvo con la duración de la LME.

## Resultados

Por 75 días continuos se invitó a participar a 453 puérperas, 15 de las cuales lo rechazaron. Se conformó una cohorte única de 438 mujeres. Después de la entrevista inicial, 24 mujeres (5.5%) no pudieron ser localizadas para la primera visita domiciliar al 8 día ni en la dirección ni en el teléfono reportado y fueron declaradas como pérdidas. Dos mujeres más (0.5%), sufrieron muerte de sus bebés antes de la primera visita. Entre la primera y la quinta visita se presentaron 12 pérdidas adicionales (2.7%), la mayoría por cambio no reportado de domicilio de la participante. El total de pérdidas durante el seguimiento fue de 38 mujeres (8.7%). La mayoría de las mujeres participantes se encontraba entre 15 y 24 años, en unión libre, con estudios de secundaria y dedicadas al hogar. La distribución del tipo de afiliación fue similar entre las participantes (Tabla 1).

**Tabla 1:** Características sociodemográficas de las mujeres de la cohorte de Cali.

Característica	n	%
Edad en años		
< 15	16	3.7
15-24	362	82.6
> 24	60	13.7
Estado civil		
Soltera	144	32.9
Casada	29	6.6
Unión libre	263	60.0
Otro	2	0.5
Escolaridad		
Ninguna	3	0.7
Primaria	70	16.0
Secundaria	317	72.4
Técnica/ Universitaria*	48	11.0
		
Ocupación		
Hogar	323	73.7
Trabajo	52	11.9
Estudios	18	4.1
Dos o más	42	9.6
		
Régimen de seguridad social		
Contributivo	144	33.0
Subsidiado	160	36.6
No reporta	133	30.4

*La escolaridad técnica/universitaria se indagó como una sola categoría

Poco más de la mitad de recién nacidos fueron de sexo masculino (50.5%), el peso al nacer presentó una distribución normal con promedio 3,297 g (IC 95%: 3,173-3,241) y la vía vaginal fue la más utilizada en el parto (n= 323, 73.7%). Respecto de la concepción y el embarazo 267 (61.0%) de las participantes manifestaron no esperar el embarazo; 344 (78.5%) de ellas conoció su embarazo antes del tercer mes de gestación y 427 (97.5%) declararon haber asistido a control prenatal. 254 (58.0%) de las participantes no recibieron orientación del personal de salud sobre LM durante el embarazo y, entre el nacimiento y el ingreso al estudio, 283 (64.6%) no habían recibido ninguna orientación. La proporción de mujeres de régimen contributivo que recibieron orientación por el personal de salud fue mayor que la proporción de afiliadas al subsidiado, y no afiliadas (Tabla 2).

**Tabla 2:** Proporción de mujeres con orientación en lactancia por personal de salud según afiliación al Sistema de General de Seguridad Social.

Afiliación	n	Orientación durante puerperio (%)*	Orientación durante embarazo (%)**
Contributivo	144	36.8	42.4
Subsidiado	160	34.8	32.1
No-afiliado	133	28.4	25.5

*(_2gl=2_=1.60; *p*= 0.449

**(_2gl=2_=12.89; *p*= 0.002

La mayoría de las mujeres manifestaron desconocer como se conserva en casa la leche extraída. También se evidenció déficit significativo en conocimientos y habilidades básicas para mantener lactancia exitosa. Con excepción de la técnica para colocar el bebe al seno, las mujeres reportaron menor déficit en conocimiento sobre frecuencia de amamantamiento y mayores déficits en extracción de leche materna, conservación de leche y duración de la lactancia al momento del puerperio inmediato cuando es inminente el inicio de la lactancia; dichas diferencias tuvieron significancia estadística (Tabla 3).

**Tabla 3:** Frecuencia de conocimientos y habilidades básicas para mantener lactancia exitosa en embarazo y puerperio en la cohorte.

Déficit en conocimientos y habilidades básicas para lactancia	Embarazo		Puerperio inmediato		p*
	n	%	n	%	
Técnica para colocar el bebe al seno	57	13.0	65	14.8	0.50
Extracción de leche materna	119	27.2	198	45.2	0.00
Conservación en casa de leche materna	195	44.6	364	83.2	0.00
Duración de lactancia exclusiva	124	28.3	265	60.6	0.00
Frecuencia de amamantamiento	145	33.2	138	31.6	0.02

* Prueba de McNemar

La mayoría (n=284, 64.8%) refirió no haber hablado con el padre del bebé sobre LM; entre 154 (35.2%) que sí lo hicieron, la opinión del padre fue positiva en 150 (98%). En la primera entrevista la mayoría (n=415, 94.7%) no conocía norma o ley que protegiera la LM. El alojamiento conjunto se observó en 92.9%. De 438 mujeres sólo 133 (30.4%) tuvo intención de LME por 6 meses, 82 (18.8%) menos de 6 meses, 64 (14.6%) por más de 6 meses y 158 (36.2%) no sabía por cuantos meses. De 437 que expresaron intención de lactar, 72.9% refirieron estar muy seguras de hacerlo, 26.7% dijeron estar medianamente seguras y 0.4% refirió no estar seguras. Al ingresar al seguimiento 432 (98.6%) ya amamantaban su bebé, 386 (89.4%) a libre demanda y 175 (40.5%) había iniciado el amamantamiento en la primera media hora posparto. Entre 185 mujeres con dificultades para amamantar, 52 (28.1%) manifestaron que no les bajaba leche, 43 (23.2%) dolor en los senos y 52 (28.1%) no sabían cómo colocar al bebé. La Figura 1 muestra la disminución progresiva y marcada de la proporción de mujeres que mantuvieron LME a medida que avanzó el seguimiento. A 8 días poco más de la mitad mantenía LME y, al final del seguimiento, esta proporción se redujo a solo 1.4%. 

**Figura 1:** Sobrevida de lactancia materna exclusiva en mujeres primíparas lactantes de Cali, Colombia.

Se observó que la tasa de incidencia de abandono de la LME en la cohorte fue de 33.77 (IC 95%: 30.50-37.39) abandonos por 1,000 mujeres-día de LME. A los 3 días (IC 95%: 3.0-4.0) 25% ya había abandonado la LME; a los 11 días (IC 95%: 7.1-13.0) 50% ya lo habían hecho y a los 44 días (IC 95%: 35.0-48.0) un 75% había abandonado la LME. Se determinó que únicamente cuatro variables entre las estudiadas se asociaron a duración de LME con nivel de significancia *p* (0.25 y fueron éstas las seleccionadas para el análisis múltiple. Se encontró que las mujeres que ya estaban lactando en las primeras 4 horas posparto, presentaron una duración de LME significativamente mayor que las que no lo hicieron. Las mujeres que expresaron estar muy seguras de lactar a sus bebés lactaron en forma exclusiva por más tiempo que las que expresaron estar medianamente o poco seguras en hacerlo. Cuando el padre del bebé tuvo una opinión positiva frente a la lactancia, la duración de LME fue significativamente mayor que cuando no la tenía o no había opinado del tema. Se encontró que en el rango de peso al nacer que se estudió la relación con la duración de la LME no fue lineal. Puesto que en los rangos de peso entre 2,500 - 3,021 g y 3,388-4,500 g se observó mayor duración de LME que cuando el recién nacido pesó entre 3,022 y 3,387 el peso del recién nacido fue categorizada teniendo en cuenta tal relación (Tabla 4). 

**Tabla 4:** Variables que influyeron en la duración de la lactancia exclusiva.

Variable	n	Mediana de LE en días	IC 95%	Log Rank Test	*p*
Seguridad para amamantar					
Muy segura	199	17	13 - 27	7.29	0.007
Mediana o nada segura	239	12	7 - 15		
Opinión positiva del padre sobre lactancia					
Si	150	17	12 - 26	4.81	0.028
No o No ha opinado	288	12	9 - 16		
Peso del recién nacido					
">≤3,021 o >3,387 g	274	15	12 - 21	4.67	0.031
3,022 a 3,387 g	164	11	7 - 16		
Lactando en puerperio					
Si	432	14	12 - 17	6.95	0.008
No	6	2	-		

La asociación de la variable que indica si durante el puerperio inmediato se estaba lactando o no, y la duración de la LME, se torna no significativa estadísticamente durante el análisis múltiple. Sin embargo, a pesar de un cambio mayor al 10% entre las estimaciones crudas y ajustadas para esta variable, no se evidenció que el cambio hubiese sido influido (confusión o modificación de efecto) por las otras variables asociadas a la duración de LME. Las demás razones de peligro crudas y ajustadas no difieren de manera significativa. Así, es plausible que la relación de cada variable asociada con duración de LME es independiente y no es influida por demás variables asociadas. En consecuencia, se esperaría que si se logra implementar una intervención que mejore la seguridad para amamantar en mujeres de similares características a las estudiadas, el riesgo de abandonar tempranamente la LME exclusiva se podría disminuir significativamente en 28%. De igual manera, si se logra que una mayor proporción de padres, similares a los compañeros de mujeres estudiadas, tengan una opinión positiva de la lactancia, se esperaría que el riesgo de abandonar tempranamente la LME se disminuya significativamente hasta en 26%. Por último, si se logra promover de manera efectiva la LME en recién nacidos de mujeres similares a las estudiadas cuyo peso esté entre 3,022-3,387 g, se esperaría que el riesgo de abandonar tempranamente la LME se disminuya significativamente en 23%. (Tabla 5)

**Tabla 5:** Razones de peligro crudas y ajustadas para abandonar lactancia exclusiva

Variable	Razones de peligro crudas	IC 95%	Razones de peligro ajustadas	IC 95%
Seguridad para dar lactancia				
Muy segura	1.00	-	1.00	-
Mediana o nada segura	1.32	1.07-1.62	1.28	1.04-1.58
Opinión positiva del padre sobre lactancia				
Si	1.00	-	1.00	-
No o No ha opinado	1.27	1.02-1.58	1.26	1.01-1.57
Peso del recién nacido				
≤3,021 or >3,387 g	1.00	-	1.00	-
3,022 a 3,387 g	1.26	1.02-1.56	1.23	1.00-1.53
Lactando en el puerperio inmediato				
Si	1.00	-	1.00	-
No	5.01	1.23-20.43	4.07	0.96-16.67

## Discusión

Este fue un estudio en una cohorte única y fija de mujeres en lactancia residentes en Cali que alcanzó su objetivo de identificar posibles factores causales de la duración de LME. Este diseño permitió la medición de la duración de LME de manera prospectiva y no retrospectiva, como en la mayoría de los estudios nacionales. La medición prospectiva le confiere ventajas puesto que los estudios que miden retrospectivamente la duración de LME introducen sesgos de información que tienen alta probabilidad de afectar la validez de los resultados ^30^^-^^32^. Las diferencias en la estimación de la duración de la LME y la proporción de mujeres que practican LME de este estudio con la Encuesta Nacional de Demografía y Salud en Colombia ^14^, probablemente se deban no solo a la diferencia que puedan existir en factores comportamentales y/o socio-culturales y de prestación de servicios de salud entre Cali y el resto del país, sino también a la validez de los métodos usados en las mediciones. La Encuesta Nacional de Demografía y Salud estima la duración de LME y la proporción de mujeres que la practican mediante técnicas retrospectivas que pretenden evocar la recordación en mujeres que amamantaron varios años atrás, lo cual afecta la validez de la estimación ^32^. 

En este estudio se encontró que la mayoría de mujeres expresaron una firme intención de amamantar en el puerperio inmediato, pero la proporción que finalmente brindó lactancia a sus bebés durante los primeros seis meses fue baja; muy pocas brindaron LME por el tiempo recomendado. Este hallazgo cuestiona uno de los supuestos fundamentales de la TCP, el cual plantea que la intención de ejecutar un comportamiento es un buen predictor de la ejecución del mismo. Sin embargo, es posible que se requiera mejorar la indagación de la intención de amamantar. 

Este estudio también evidenció que factores presentes antes del parto y en el puerperio inmediato están involucrados de manera independiente en la duración de la LME. El efecto del inicio de la lactancia en el puerperio inmediato sobre duración de LME ya ha sido encontrado en otras investigaciones. El mecanismo mediante el cual este factor se involucra en la duración de LME es al parecer porque la succión temprana favorece la lactogénesis estadio dos o “bajada de la leche” y es cuando se establece la producción continua de leche y se tiene capacidad para responder a demandas del recién nacido. Esta variable muestra una asociación significativa en el análisis bivariado (p=0.008) que posteriormente se torna no significante en el análisis múltiple, posiblemente por la baja frecuencia de mujeres que no iniciaron tempranamente LM. Sin embargo, a pesar del amplio intervalo de confianza, se incluye marginalmente el valor nulo de la razón de peligro ajustada. Esta situación no descarta el efecto de esta variable sobre LME en Cali y para futuros estudios, se debe seguir teniendo en cuenta puesto que puede llegar a producir ajustes en las relaciones de otras variables con LME (*p* <0.25 en bivariado), si se aumenta la cantidad de mujeres que no inician tempranamente LM como se puede esperar si las tendencias de duración y practica de LME reportadas por las encuestas nacionales son válidas.

Este estudio encontró que la autopercepción de seguridad frente al inicio y mantenimiento de la LME en el puerperio inmediato está involucrada en la duración ^16^^,^^33^. Esta información obtenida de mujeres primíparas residentes en Cali, sin experiencia práctica en lactancia, sugiere que en la causalidad de la duración de LME están implicados factores del puerperio inmediato. De acuerdo con la TCP, una mediana o poca seguridad auto-percibida desencadena una sucesión de eventos que finalmente no favorecen la práctica de LME. En primer lugar, la baja auto-percepción induce en la mujer la sensación de que no va a controlar la lactancia y, por tanto, no podrá sortear las barreras para amamantar o utilizar a su favor condiciones que la faciliten. Por consiguiente, se debilita la intención de amamantar, se recurre a sucedáneos de la leche materna con mayor facilidad y se interrumpe LME. 

Sin embargo, se ha encontrado que en la medida que avanza la lactancia, la seguridad para amamantar en las primíparas se va elevando hasta alcanzar los mismos niveles de multíparas. Este es un posible mecanismo mediante el cual la red de soporte social (especialmente padre y abuelas del bebé) prolonga la duración de la lactancia. El efecto de la opinión del padre del bebé sobre la duración de la LME encontrado en este estudio coincide con los resultados de otros estudios ^20^^,^^34^ y lo sitúa como uno de los potenciales actores que podría apoyar la prolongación y mantenimiento de la LME en poblaciones similares a la estudiada. Desde la TCP, la opinión positiva del padre eleva la percepción de que muchas personas aprueban la LME en la mujer, puesto que se establece en ellas la sensación de que este referente inmediato aprueba la LME y se aumenta la motivación para complacerlo. Así, se refuerza la intención de brindar LME y efectivamente la practican por más tiempo. En consecuencia, cuando el padre del recién nacido es entrenado para que brinde un mejor apoyo a la mujer que amamanta, se prolonga por más tiempo LME y se eleva la proporción de mujeres que la practican ^20^^,^^34^. 

La relación entre peso al nacer y duración de LME encontrada concuerda parcialmente con los resultados de estudios prospectivos ^16^^,^^35^^,^^36^. Coincide en que se continúa observando que los niños de menor peso al nacer son los que reciben LME por más tiempo. Pero que los recién nacidos de peso mayor a 3,387 g reciban LME por más tiempo es un hallazgo particular. Una posible explicación es que los profesionales de salud que atendieron a las mujeres estudiadas brindaron mayor apoyo teórico-práctico en lactancia a mujeres cuyos recién nacidos pesaron 3,021 g o menos, o cuando pesaron más de 3,387 g, en busca de una ganancia de peso más rápida en los primeros y evitar una potencial hipoglicemia en los segundos. Esta asociación debe ser abordada en estudios posteriores. 

La información y soporte brindado durante el embarazo y el puerperio no fueron involucrados en la duración de la LME. Es posible que en una población como la estudiada, en donde la duración de la LME es tan corta y sea practicada durante 6 meses por tan pocas mujeres, el alto nivel de desconocimiento en aspectos básicos no presente un gradiente suficiente como para establecer diferencias. Otra posibilidad es que durante el transcurso de la lactancia el déficit de conocimientos se corrija y/o surjan nuevos factores o se modifiquen los factores presentes en el embarazo o en el puerperio y sean estos los que más expliquen el abandono de la LME. Esta última posibilidad no puede ser soportada con este estudio porque la metodología se centró en factores presentes en el embarazo y en el puerperio inmediato, y no se midió la evolución que estos factores pudieron haber tenido en el transcurso del seguimiento. Esta debilidad debe ser abordada en estudios prospectivos posteriores. 

Factores como escolaridad materna, estrato socio-económico, ocupación, estado civil y tipo de parto, variables que tradicionalmente han sido involucradas a duración de LME, no estuvieron implicadas en la duración de LME en Cali. Esto refleja una homogeneidad en la distribución de estas variables socio-económicas entre mujeres que abandonaron tempranamente la LME y las que no lo hicieron. Con respecto a la vía de parto, es posible que no esté involucrada en la duración de LME en esta población porque en la gran mayoría el parto fue vaginal (73.7%) y casi todas las mujeres y sus bebés tuvieron alojamiento conjunto e iniciaron lactancia tempranamente, contrarrestando el efecto de la cesárea.

Los binomios madre-niño afiliados a los regímenes del Sistema de Salud no presentaron diferencias en duración de LME con respecto a los binomios no afiliados al sistema. Sin embargo, la proporción de mujeres que recibieron orientación durante el embarazo fue significativamente menor en afiliadas al régimen subsidiado y en las no afiliadas con respecto a afiliadas al régimen contributivo. Esto sugiere desigualdades en cuanto a la prestación de servicios según el tipo afiliación. Este hallazgo insinúa poca efectividad del Sistema de Salud para promover la lactancia porque, a pesar de ofrecerle más educación y acompañamiento a las mujeres afiliadas al régimen contributivo, no logra que la duración sea mayor que la observada en las subsidiadas o no-afiliadas. 

## Conclusiones

Existen factores individuales, familiares y de los servicios de salud presentes antes e inmediatamente después del parto que limitan la duración de LME en Cali.

La utilización de la TCP fue conveniente para identificar posibles factores causales de la corta duración de LME en Cali y orientó sobre las posibles vías por donde se desencadenan sus efectos. 

**Recomendaciones**. Es prioritario elevar el conocimiento y mejorar las prácticas sobre aspectos básicos de lactancia, no solo en las mujeres embarazadas o en puerperio, sino en las personas más significativas e influyentes para estas mujeres, como son los padres de los recién nacidos. También, se deben reforzar las habilidades para educar en lactancia al personal institucional. 

La seguridad para amamantar, la opinión que el padre del recién nacido tiene de la lactancia y el peso al nacer pueden ser utilizados de tres maneras para abordar la corta duración de LME. En primer lugar, modificar estos factores deben ser el objetivo de las intervenciones que se implementen desde el puerperio inmediato. En segundo lugar, los factores identificados pueden ayudar a detectar en el puerperio inmediato las mujeres a mayor riesgo de abandonar tempranamente la LME e iniciar oportunamente una intervención en ellas. Por último, la medición de la duración de la LME en este estudio puede servir de referencia para comparar la efectividad de diferentes alternativas de intervención tendientes a prolongar la duración de LME.
